# Hazard rate estimation when the measurement error has a normal or logistic distribution

**DOI:** 10.1016/j.heliyon.2024.e27730

**Published:** 2024-03-13

**Authors:** Parviz Nasiri, Rougheih Kheirazar, Abbas Rasouli, Ali Shadrokh

**Affiliations:** a Department of Statistics, University of Payam Noor, 19395-4697, Tehran, Iran; b Department of Statistics, University of Zanjan, Zanjan, Iran

**Keywords:** Kernel density estimation, Bandwidth, Logistic distribution, Lifetime data, Mean squared error

## Abstract

Statistical data analysis available in most scientific fields is often recorded with measurement error. The modeling of these statistical data by ignoring the measurement errors, leads to estimators of the parameters of the distributions, whose use does not achieve sufficient accuracy in the goodness of fit. In reliability criteria, one of the important issues is hazard rate function. It prompted us to investigate the hazard rate criterion in the presence of measurement error generated from the normal or logistic distribution. Now, while providing the estimator for the density function using local time polynomial estimator methods, the risk rate function is estimated according to the contamination degree of 15 or 30%. Finally, we present the numerical analysis.

## 1 Introduction

In the analysis of statistical data that includes measurement error, the calculation of reliability measures, including the hazard rate function, is very importance. Analyzing and modeling these types of data, by ignoring measurement errors, would increase the bias of estimators. Let *T* be a lifetime random variable such that F(t)=P(T≤t) then hazard rate function is given by:

(1.1) $$\lambda \left(t\right)=\underset{\Delta \to 0}{\lim}\;\frac{P\left(t\leq T<t+\Delta \right)}{\Delta P(T>t)}=\frac{f(t)}{\overset{¯}{F}(t)},$$

and λ(t)=0 whenever $\overset{¯}{F}(t)=0$. For more details see [1]. One of the important factors in the analysis of statistical data in various fields such as medicine, economics, astronomy, etc. is the measurement error investigation. Ignoring measurement error in this type of data can lead to biased estimates and wrong conclusions (see [2], [3] and [4]). In this paper, the parameter λ(t) is estimated when the statistical data have measurement error generated from the normal or logistic distribution.

It is important to provide an estimator for the hazard rate function or equation (1.1). For this purpose, a non-parametric framework is considered to estimate the hazard rate function (see [5], [4] and [6]).

One of the suggested estimators is the use of local polynomial regression (see [7], [8], [9], [10] and [11]). In modeling data in the presence of measurement error, the main problem is the unavailability of real observations, such that instead of their observations, independent observations $Y_{1},Y_{2},\ldots ,Y_{n}$ are available. Therefore, the following model is considered.

(1.2) Y=X+ε.

Such that, for simplicity, it is assumed that the random variable *ε* has a normal distribution with a mean of zero and a variance of $\sigma_{\varepsilon }^{2}$. In this collective model, to estimate the density function of the random variable *X*, according to the distribution of *ε* and the observed values for the random variable *Y*, the density function of random variable *X* can be estimated by the well-known deconvolution estimator as (See [3], [4] and [12]):

(1.3) $${\overset{ˆ}{f}}_{X}\left(x\right)=\frac{1}{2\pi }\int \exp\left(-itx\right)\phi_{K}\left(tb\right)\frac{\frac{1}{n}\sum_{j=1}^{n}\exp\left(itY_{j}\right)}{\phi_{\varepsilon }\left(t\right)}dt=\frac{1}{n}\sum_{j=1}^{n}\frac{1}{2\pi }\int e^{it\left(Y_{j}-x\right)}\frac{\phi_{K}\left(tb\right)}{\phi_{\varepsilon }\left(t\right)}dt,$$

where $\phi_{K}$ and $\phi_{\varepsilon }$ are characteristic functions of *K* and *ε*, respectively. It is easy to show that the characteristic function of normal and logistic distribution given by:

$$\phi_{X}(t)=\exp(i\mu t-\frac{\sigma^{2}t^{2}}{2})\;and\;\phi_{X}(t)=e^{i\mu t}\frac{\pi st}{\sin h(\pi st)}$$

respectively.

### 1.1 Plug-in estimator

As we know observed or recorded values for lifetime data are positive values even with measurement errors. Providing a collective model in this case may produce negative values. To avoid this, it is necessary to provide a multiplicative model. Therefore, to avoid taking negative values of the variables, the multiplicative model

(1.4) $$Z_{i}=T_{i}\delta_{i},\;i=1,\ldots ,n,$$

is considered. But it can be easily shown that

(1.5) $$Y_{i}=X_{i}+\varepsilon_{i},\;i=1,\ldots ,n.$$

Such that $Y_{i}=\ln(Z_{i})$, $X_{i}=\ln(T_{i})$ and $\varepsilon_{i}=\ln(\delta_{i})$. Now to estimation of $\overset{ˆ}{f}(.)$, we can write:

$$F_{X}(t)=P(T\leq t)=P(\ln(T)\leq \ln(t))=P(X\leq \ln(t)),$$

such that $F_{X}^{\prime }(t)$ given by:

(1.6) $$\overset{ˆ}{f}\left(t\right)=\frac{1}{t}{\overset{ˆ}{f}}_{X}\left(\ln(t)\right).$$

Now, by using the (1.6), the risk rate function estimator can be considering as:

(1.7) $$\overset{ˆ}{\lambda }\left(t\right)=\frac{\overset{ˆ}{f}\left(t\right)}{\int_{t}^{\tau_{F}}\overset{ˆ}{f}\left(x\right)dx}=\frac{{\overset{ˆ}{f}}_{X}\left(\ln\left(t\right)\right)}{t\left[1-{\overset{ˆ}{F}}_{X}\left(\ln\left(t\right)\right)\right]}.$$

Note that in the case where the errors are non-random and systematic, the above problem can be considered as the estimation of the hazard rate function under biased samples which has received attention by some researches (cf. [13]).

### 1.2 Local polynomial estimator

Let $x_{1},...,x_{m}$ be *m* selected points from random sample $X_{1},X_{2},...,X_{n}$. For selecting these points on a sub set $\left[0,\tau_{F_{X}}\right]$ with $\tau_{F_{X}}:=\inf\{x:F_{X}(x)=1\}$, we partition the interval into *m* disjoint subintervals $\left\{I_{i},i=1,...,m\right\}$ of equal length $\Delta =\tau_{F_{Y}}/m$, and let the $x_{i}$ be the center of each bin $I_{i}$. For the number of observations $f_{i}$ in the interval $I_{i}$ the local polynomial estimator of the rate function is given by (See [2], [14] and [15]):

(1.8) $$\overset{ˆ}{\lambda }\left(x_{i}\right)=\frac{f_{i}}{\Delta \left(n-\sum_{j=1}^{i-1}f_{j}+1\right)},\;\;\;\;\;i=1,2,...,m.$$

Based on [16], the local polynomial estimator of order the hazard rate function approximates given by,

$$\overset{ˆ}{\lambda }(z)=\sum_{j=1}^{p}\frac{{\overset{ˆ}{\lambda }}^{\left(j\right)}(x_{0},p)}{j!}{\left(z_{i}-x_{0}\right)}^{j}=\frac{{\overset{ˆ}{\lambda }}^{\left(1\right)}(x_{0},p)}{1!}(z_{1}-x_{0})+\frac{{\overset{ˆ}{\lambda }}^{\left(2\right)}(x_{0},p)}{2!}{\left(z_{2}-x_{0}\right)}^{2}+...+\frac{{\overset{ˆ}{\lambda }}^{\left(p\right)}(x_{0},p)}{p!}{\left(z_{p}-x_{0}\right)}^{p}$$

Such that $Z_{i}=X_{i}+\epsilon_{i}$, $\epsilon_{i}∼N(0,\sigma_{\epsilon }^{2})$. For

$$\beta_{j}=\frac{{\overset{ˆ}{\lambda }}^{\left(j\right)}(x_{0},p)}{j!}\;\text{and}\;\overset{ˆ}{\lambda }(z)=\sum_{j=1}^{p}\beta_{j}{\left(z_{i}-x_{0}\right)}^{j}$$

the estimator of $\overset{ˆ}{\beta }={\left({\overset{ˆ}{\beta }}_{1},{\overset{ˆ}{\beta }}_{2},...,{\overset{ˆ}{\beta }}_{p}\right)}^{T}$ can be obtained by minimization of:

$$\sum_{i=1}^{m}{\left[\overset{ˆ}{\lambda }\left(x_{i}\right)-\sum_{j=0}^{p}\beta_{j}{\left(x_{i}-x_{0}\right)}^{j}\right]}^{2}K_{b}\left(x_{i}-x_{0}\right),$$

where $K_{b}(.)=b^{-1}K(./b)$, *K* a kernel function and *b* is the bandwidth. The local polynomial estimator $\overset{ˆ}{\beta }={\left({\overset{ˆ}{\beta }}_{0},...,{\overset{ˆ}{\beta }}_{p}\right)}^{T}$ with ${\overset{ˆ}{\beta }}_{j}={\overset{ˆ}{\lambda }}^{\left(j\right)}(x_{0};p)/j!$ is:

(1.9) $$\overset{ˆ}{\beta }={\left({\text{X}}^{T}\text{W}\text{X}\right)}^{-1}{\text{X}}^{T}\text{W}\Lambda ,$$

where, $\Lambda =(\overset{ˆ}{\lambda }(x_{1}),\overset{ˆ}{\lambda }(x_{2}),...,\overset{ˆ}{\lambda }(x_{m}))\;,\;W=diag{\left(k_{b}(x_{i}-x_{0})\right)}_{m\times m}$.

Hence, the estimator for the $\lambda (x_{0})$ of order *p*, is given by ${\overset{ˆ}{\beta }}_{0}$, i.e.:

(1.10) $$\overset{ˆ}{\lambda }(x_{0})={\text{e}}_{1}^{T}{\left({\text{X}}^{T}\text{W}\text{X}\right)}^{-1}{\text{X}}^{T}\text{W}\Lambda .$$

Here according to the type of data and using the multiplicative model and contaminated data, our goal present new estimator by replacing the $L^{⁎}(z_{i}-x_{0})$ satisfying (See [5], [17] and [18]):

$$E\left(L^{⁎}\left(Z_{i}-x_{0}\right)|X_{i}\right)=K_{b}\left(X_{i}-x_{0}\right),$$

where $L^{⁎}(u)=L_{1}(-u)$. Following this idea and from [19], our new estimator will be as follows:

(1.11) $$\overset{ˆ}{\beta }={\left({\text{Z}}^{T}\text{L}\text{Z}\right)}^{-1}{\text{Z}}^{T}\text{L}\Lambda ,$$

and our new suggested estimator for the λ(x) of order *p*, is given by ${\overset{ˆ}{\beta }}_{0}$, i.e.:

(1.12) $$\overset{ˆ}{\lambda }(x_{0};p)={\text{e}}_{1}^{T}{\left({\text{Z}}^{T}\text{L}\text{Z}\right)}^{-1}{\text{Z}}^{T}\text{L}\Lambda .$$

Such that $\text{L}=diag{\left(L_{b}^{⁎}\left(z_{i}-x_{0}\right)\right)}_{m\times m}$ and $\overset{ˆ}{\lambda }(z_{i})$, i=1,...,m are (1.7) estimators related to *m* selected points.

According to the estimators $\overset{ˆ}{\lambda }\left(t\right)=\frac{\overset{ˆ}{f}\left(t\right)}{\int_{t}^{\tau_{F}}\overset{ˆ}{f}\left(x\right)dx}=\frac{{\overset{ˆ}{f}}_{X}\left(\ln\left(t\right)\right)}{t\left[1-{\overset{ˆ}{F}}_{X}\left(\ln\left(t\right)\right)\right]}$ and $\overset{ˆ}{\lambda }(x_{0},p)=e_{1}^{T}{\left(Z^{T}LZ\right)}^{-1}Z^{T}L\Lambda$, in the rest of this section, the features and asymptotic properties of the estimators are discussed (See [20]).

## 2 Asymptotic results

In this section, the behavior of the risk rate function estimator is investigated using Theorems 1 and 2 of [21]. In the following, it is shown that the distribution of the hazard rate function estimator is asymptotically normal.

### 2.1 Asymptotic results for the plug-in estimator

To present the asymptotic properties of the hazard rate function estimator according to the density function *f*, random variables *T* and the characteristic distribution of the measurement error

(2.1) $$\left|\phi_{\varepsilon }\left(t\right)\right|>0,\;\;\;for\;all\;t\in R.$$

For $K(x)=\frac{1}{2\pi }\int e^{-itx}\phi_{K}(t)dt$ and fixed positive b satisfying

(2.2) $$\underset{t\in R}{\mathrm{Sup}}\;\left|\phi_{K}\left(t\right)/\phi_{\varepsilon }\left(\frac{t}{b}\right)\right|<\infty ,\;\int \left|\phi_{K}\left(t\right)/\phi_{\varepsilon }\left(\frac{t}{b}\right)\right|dt<\infty .$$

### 2.2 Asymptotic results for the local polynomial estimator

Based on local polynomial estimator of $\overset{ˆ}{\beta }$ in the equation (1.11) to obtain the $var\{\overset{ˆ}{\lambda }(x_{0},p)\}$ we can write:

(2.3) $$E\left(\overset{ˆ}{\beta }\right)={\left({\text{Z}}^{T}\text{L}\text{Z}\right)}^{-1}{\text{Z}}^{T}\text{L}\text{m}=\beta +{\left({\text{Z}}^{T}\text{L}\text{Z}\right)}^{-1}{\text{Z}}^{T}\text{L}\text{r}\;Var\left(\overset{ˆ}{\beta }\right)={\left({\text{Z}}^{T}\text{L}\text{Z}\right)}^{-1}\left({\text{Z}}^{T}\Sigma \text{Z}\right){\left({\text{Z}}^{T}\text{L}\text{Z}\right)}^{-1},$$

where $\text{m}={\left\{E\overset{ˆ}{\lambda }(z_{1}),...,E\overset{ˆ}{\lambda }(z_{m})\right\}}^{T}$, r=m−Zβ and $\Sigma =diag(L_{b}^{⁎2}(z_{i}-x_{0})var(\overset{ˆ}{\lambda }(z_{i})))$.

These exact bias and variance expressions are not directly usable, since they depend on unknown quantities. Hence, there is a need for approximating bias and variance. The following notation will be used:

$$\begin{array}{c} \mu_{j}=\int u^{j}L^{⁎}\left(u\right)du,\;\upsilon_{j}=\int u^{j}L^{⁎2}\left(u\right)du\\ \text{S}={\left(\mu_{j+\ell }\right)}_{\left(p+1\right)\times \left(p+1\right)},\;\;\;\;\;j,\ell =0,1,...,p\\ \overset{˜}{\text{S}}={\left(\mu_{j+\ell +1}\right)}_{\left(p+1\right)\times \left(p+1\right)},\;\;\;\;\;j,\ell =0,1,...,p\\ {\text{S}}^{⁎}={\left(\upsilon_{j+\ell }\right)}_{\left(p+1\right)\times \left(p+1\right)},\;\;\;\;\;j,\ell =0,1,...,p\\ {\text{C}}_{p}={\left(\mu_{p+1},...,\mu_{2p+1}\right)}^{T}\;{\overset{˜}{\text{C}}}_{p}={\left(\mu_{p+2},...,\mu_{2p+2}\right)}^{T}. \end{array}$$

According to the notations, the variance and bias of the estimator $\overset{ˆ}{\lambda }(x_{0},p)$ are respectively equal to:

(2.4) $$Var\left\{\overset{ˆ}{\lambda }\left(x_{0};p\right)\right\}={\text{e}}_{1}^{T}{\text{S}}^{-1}{\text{S}}^{⁎}{\text{S}}^{-1}{\text{e}}_{1}\frac{\sigma^{2}\left(x_{0}\right)}{f_{Y}\left(x_{0}\right)nmb}+o_{P}\left(\frac{1}{nmb}\right)$$

For *p* odd

(2.5) $$Bias\left\{\overset{ˆ}{\lambda }\left(x_{0};p\right)\right\}={\text{e}}_{1}^{T}{\text{S}}^{-1}{\text{C}}_{p}\frac{h^{p+1}\lambda^{\left(p+1\right)}\left(x_{0}\right)}{\left(p+1\right)!}+o_{P}\left(h^{p+1}\right),$$

and for *p* even

(2.6) $$Bias\left\{\overset{ˆ}{\lambda \left(x_{0};p\right)}\right\}={\text{e}}_{1}^{T}{\text{S}}^{-1}{\overset{˜}{\text{C}}}_{p}\frac{h^{p+2}}{\left(p+2\right)!}\left\{\lambda^{\left(p+2\right)}\left(x_{0}\right)+\left(p+2\right)\lambda^{\left(p+1\right)}\left(x_{0}\right)\frac{f_{Y}^{\left(1\right)}\left(x_{0}\right)}{f_{Y}\left(x_{0}\right)}\right\}+o_{P}\left(h^{p+2}\right).$$

Provided that $f_{Y}^{\left(1\right)}(.)$ and $\lambda^{p+2}(.)$ are continuous in a neighborhood of $x_{0}$.

## 3 Bandwidth selection

One of the methods to reduce the mean square error in the estimation of the usage rate function is to select the bandwidth. So the selection of the bandwidth in deconvolution problems has recently attracted the attention of a number of researchers, for more information, you can refer to articles [22], [23], [24] and [25]. In Section 3.1, a rule of thumb has been used to select the bandwidth when the measurement error has a normal or logistic distribution (see [26] and [27]).

### 3.1 Rule of thumb

In this section, according to the estimation methods of the risk rate function, the criterion (MISE) is used to determine the bandwidth as

$$MISE\{\overset{ˆ}{f}\}=E\left[\int {\left(\overset{ˆ}{f}(x)-f(x)\right)}^{2}dx\right].$$

According to and the characteristic function of the measurement error, the optimal value of the bandwidth is given by (See [27])

$$b_{ROT,N}=\sqrt{2}\sigma_{\varepsilon }{\left(\log n\right)}^{-1/2},$$

and based on rule of thumb bandwidth in case of ordinary smooth the value of the bandwidth is given by:

$$b_{ROT,L}={\left(\frac{5\sigma_{\varepsilon }^{4}}{n}\right)}^{1/9}.$$

## 4 Simulation study

In this section, the main goal is to compare the obtained estimators with the estimator [28], that is, the plug-in estimator.

(4.1) $${\overset{ˆ}{\lambda }}_{C}\left(x_{0}\right)=\frac{\overset{ˆ}{f}\left(x_{0}\right)}{1-{\overset{ˆ}{F}}_{\lambda }\left(x_{0}\right)},$$

where $\overset{ˆ}{f}$ is defined in (1.3) and ${\overset{ˆ}{F}}_{\lambda }$ is the estimator introduced by [29], i.e.

(4.2) $${\overset{ˆ}{F}}_{\lambda }\left(x_{0}\right)=\frac{1}{2}-\frac{1}{n}\sum_{j=1}^{n}\frac{1}{\pi }\int_{0}^{\lambda }\frac{1}{\omega }ℑ\left\{\frac{e^{i\omega \left(Y_{j}-x_{0}\right)}}{\phi_{\varepsilon }\left(\omega \right)}\right\}d\omega$$

Comte et al. [28] discussed the value of *λ* in (4.2). We can use [30] results in the Theorem 1 and the Theorem 2 for determination of *λ*, but for convenience here we take $\lambda =\sqrt{n}$ from the page 9 of [29].

Now to compare the risk rate function estimation methods according to the amount of contamination produced from the normal or logistic distribution, the results for Weibull, Gamma, and Lindley distributions are evaluated. In the simulation part, we use the second-order kernel function to estimate the density function corresponding to the hazard rate function under contaminated data (see [24], [31] and [32]),

(4.3) $$K(x)=\frac{48\cos x}{\pi x^{4}}\left(1-\frac{15}{x^{2}}\right)-\frac{144\sin x}{\pi x^{5}}\left(2-\frac{5}{x^{2}}\right)$$

with characteristic function

$$\phi_{K}\left(t\right)={\left(1-t^{2}\right)}^{3}I_{\left[-1,1\right]}\left(t\right).$$

For the local polynomial estimator, we take m=25 and according to [33] recommendation, p=1. To determine the quantity *m*, some approaches can be found in references such as [34], [35] and [16]. For a practical and complete discussion on determining *m*, refer to section 4.1 of [36]. For selecting points $z_{1},...,z_{25}$, we let $\Delta =\tau_{F_{Y}}/25$ where $\tau_{F_{Y}}:=\inf\{y:F_{Y}(y)=1\}<\infty$ and $z_{i}=(i-0.5)\Delta$, i=1,...,25. Such a style selection fixed points for local polynomial estimator has been used in various references such as in [16], [37] and [38].

**Figure 1:**
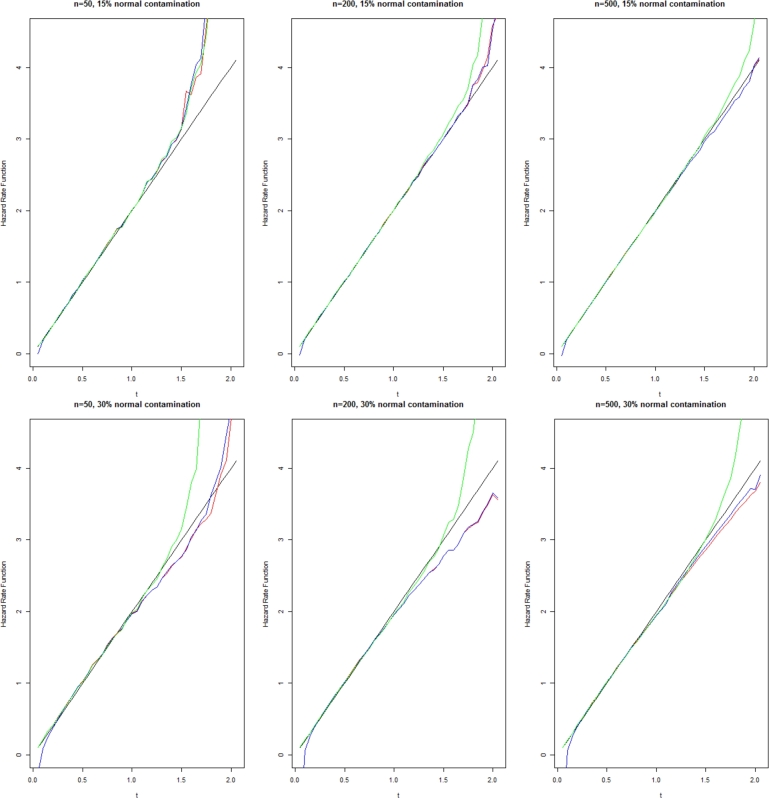
Estimator $\overset{ˆ}{\lambda }(t)$ for Weibull distribution with shape 2 and scale 1.

**Figure 2:**
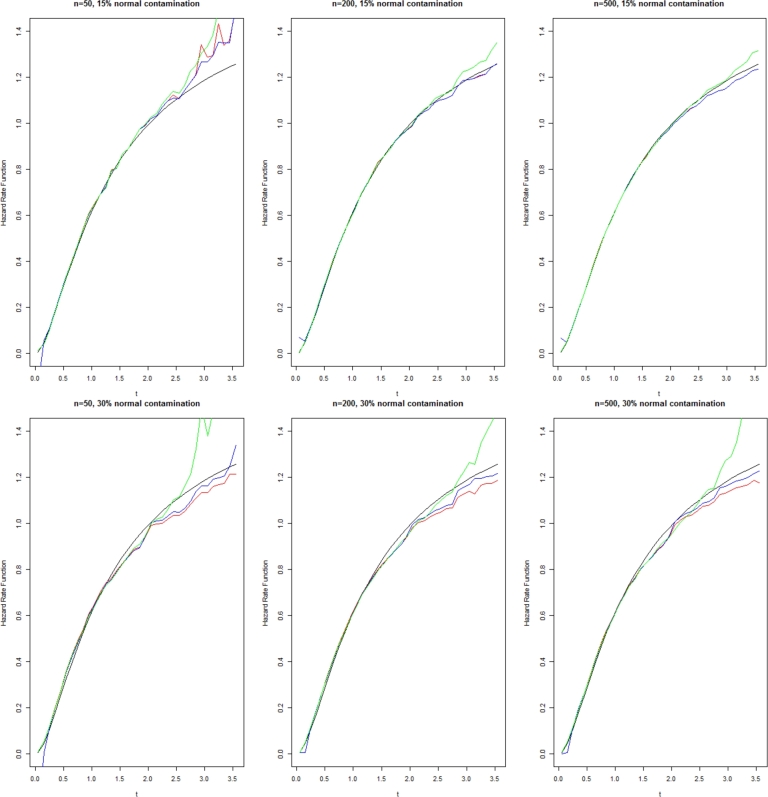
Estimator $\overset{ˆ}{\lambda }(t)$ for Gamma distribution with shape 3 and scale $\sqrt{3}$.

**Figure 3:**
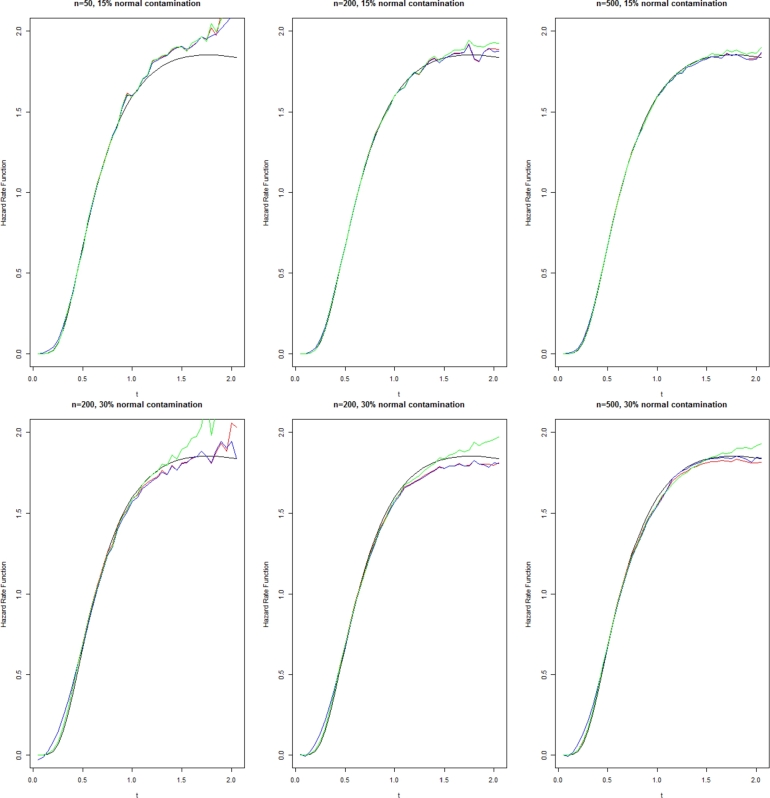
Estimator $\overset{ˆ}{\lambda }(t)$ for Lindley distribution with shape 0 and scale 0.5.

Here to compare the estimators $\overset{ˆ}{\lambda }(t,p),\overset{ˆ}{\lambda }(t)$ and ${\overset{ˆ}{\lambda }}_{C}(t)$ according to the parameters of Weibull, Gamma and Lindley distributions with 15 and 30 percent contamination generated from normal or logistic, the simulation results for the size of samples n=10,50,100 with 1000 repetitions using R software are given in Table 1, Table 2, Table 3, Table 4, Table 5, Table 6, Table 7, Table 8, Table 9, Table 10, Table 11, Table 12. Also, to compare the convenience of the behavior of the estimator, the diagram of the mean square error of estimator is given in Figure 1, Figure 2, Figure 3, such that:

**Table 1:** Mean and Mean Square Error of Hazard rate function estimators of **Weibull** distribution with exception %15 contamination **Normal error**.

		*t*				
n	Estimator	0.25	0.5	0.75	1.00	1.25

10	$\overset{ˆ}{\lambda }(t,p)$	0.5494	0.0641	1.6238	2.5925	4.4401
		(1.0017)	(1.2724)	(1.6807)	(1.9531)	(1.7560)
	$\overset{ˆ}{\lambda }(t)$	1.2226	1.0735	1.6312	2.3995	4.5371
		(0.4175)	(0.6166)	(1.3629)	(1.7428)	(1.1562)
	${\overset{ˆ}{\lambda }}_{C}(t)$	0.5909	1.0705	1.6525	2.0688	2.5194
		(1.0183)	(1.3766)	(1.7349)	(2.3920)	(3.5748)

50	$\overset{ˆ}{\lambda }(t,p)$	0.4788	1.0203	1.5230	2.0462	2.4447
		(0.1745)	(0.2346)	(0.3012)	(0.5097)	(0.8055)
	$\overset{ˆ}{\lambda }(t)$	0.4901	1.0119	1.5231	2.0428	2.4601
		(0.0789)	(0.1061)	(0.1907)	(0.4007)	(0.7450)
	${\overset{ˆ}{\lambda }}_{C}(t)$	0.4789	1.0197	1.5159	3.0494	2.4878
		(0.1796)	0.2360)	(0.3023)	(0.5140)	(0.8263)

100	$\overset{ˆ}{\lambda }(t,p)$	0.5079	1.022	1.4969	2.0076	2.5387
		(0.0901)	(0.1214)	(0.1452)	(0.2397)	(0.4436)
	$\overset{ˆ}{\lambda }(t)$	0.5093	1.0180	1.5025	2.0049	2.5313
		(0.0402)	(0.0592)	(0.0884)	(0.1753)	(0.3755)
	${\overset{ˆ}{\lambda }}_{C}(t)$	0.5076	1.0216	1.4939	2.0062	2.5605
		(0.0909)	(0.1288)	(0.31452)	(0.2412)	(0.4764)

**Table 2:** Mean and Mean Square Error of Hazard rate function estimators of **Weibull** distribution with exception %30 contamination **Normal error**.

		*t*				
n	Estimator	0.25	0.5	0.75	1.00	1.25

10	$\overset{ˆ}{\lambda }(t,p)$	0.5517	1.1122	1.6487	2.2574	3.3497
		(1.6143)	(0.8427)	(1.1671)	(4.6060)	(18.6593)
	$\overset{ˆ}{\lambda }(t)$	0.5462	1.1029	1.6423	2.2568	4.0570
		(0.1745)	(0.3855)	(0.7714)	(2.3980)	(21.6651)
	${\overset{ˆ}{\lambda }}_{C}(t)$	0.5521	1.1166	1.6441	2.1131	2.4317
		(0.6034)	(0.8409)	(1.0929)	(1.2837)	(1.5235)

50	$\overset{ˆ}{\lambda }(t,p)$	0.5154	1.0227	1.4874	1.9845	2.4582
		(0.1149)	(0.1293)	(0.1603)	(0.3029)	(0.4577)
	$\overset{ˆ}{\lambda }(t)$	0.5064	1.0316	1.4887	1.9795	2.4661
		(0.0327)	(0.0584)	(0.0668)	(0.2262)	(0.4432)
	${\overset{ˆ}{\lambda }}_{C}(t)$	0.5150	1.0193	1.4772	1.9901	2.5216
		(0.1149)	0.1295)	(0.1589)	(0.3034)	(0.4743)

100	$\overset{ˆ}{\lambda }(t,p)$	0.5240	1.0219	1.5241	1.9749	2.4268
		(0.0600)	(0.0620)	(0.0966)	(0.1270)	(0.2125)
	$\overset{ˆ}{\lambda }(t)$	0.5158	1.0173	1.5132	1.9728	2.4295
		(0.0152)	(0.0272)	(0.0609)	(0.0930)	(0.1797)
	${\overset{ˆ}{\lambda }}_{C}(t)$	0.5232	1.0171	1.5156	1.9783	2.4964
		(0.0597)	(0.0616)	(0.0971)	(0.1338)	(0.2607)

**Table 3:** Mean and Mean Square Error of Hazard rate function estimators of **Weibull** distribution with exception %15 contamination **Logistics error**.

		*t*				
n	Estimator	0.25	0.5	0.75	1.00	1.25

10	$\overset{ˆ}{\lambda }(t,p)$	1.2475	1.6142	1.5291	1.6120	1.7512
		(2.8970)	(54.7680)	(9.1122)	(4.2154)	(3.1250)
	$\overset{ˆ}{\lambda }(t)$	1.3042	1.0813	1.9171	2.1037	2.5712
		(27.3542)	(14.1097)	(36.0151)	(41.1235)	(43.1253)
	${\overset{ˆ}{\lambda }}_{C}(t)$	0.5913	1.2351	1.2101	1.3521	1.4125
		(3.9535)	(5.0403)	(5.1270)	(4.0751)	(2.1253)

50	$\overset{ˆ}{\lambda }(t,p)$	1.0611	1.0231	0.9506	1.0235	1.1202
		(0.5204)	(0.2806)	(0.2360)	(0.1234)	(2.1350)
	$\overset{ˆ}{\lambda }(t)$	0.3571	0.5323	0.7874	0.8512	2.1025
		(0.3800)	(0.8197)	(0.9120)	(0.9812)	(0.7230)
	${\overset{ˆ}{\lambda }}_{C}(t)$	0.3553	0.9566	0.9178	0.8512	0.7123
		(0.0710)	(0.2607)	(0.2606)	(0.2310)	(1.0123)

100	$\overset{ˆ}{\lambda }(t,p)$	1.0920	0.9968	0.9147	0.9012	0.8123
		(0.2542)	(0.1266)	(0.1122)	(0.0825)	(0.1631)
	$\overset{ˆ}{\lambda }(t)$	0.3801	0.7344	0.4268	0.3851	0.3120
		(15.2798)	(35.0121)	(1.1560)	(0.0514)	(0.0412)
	${\overset{ˆ}{\lambda }}_{C}(t)$	0.3577	0.9351	0.9070	1.1253	1.1012
		(0.0328)	(0.1147)	(0.0920)	(0.0132)	(0.0123)

**Table 4:** Mean and Mean Square Error of Hazard rate function estimators of **Weibull** distribution with exception %30 contamination **Logistics error**.

		*t*				
n	Estimator	0.25	0.5	0.75	1.00	1.25

10	$\overset{ˆ}{\lambda }(t,p)$	0.9485	0.6110	0.4774	0.4397	0.4102
		(0.0622)	(0.0216)	(0.0144)	(0.0122)	(0.0109)
	$\overset{ˆ}{\lambda }(t)$	0.2517	0.2531	0.2643	0.2654	0.2678
		(0.0087)	(0.0076)	(0.0087)	(0.0078)	(0.0076)
	${\overset{ˆ}{\lambda }}_{C}(t)$	0.4512	0.7195	0.5286	0.4997	0.4622
		(2.5360)	(3.5400)	(0.1085)	(0.1022)	(0.0734)

50	$\overset{ˆ}{\lambda }(t,p)$	1.0055	0.6933	0.5504	0.5099	0.4805
		(0.0210)	(0.0092)	(0.0057)	(0.0053)	(0.0040)
	$\overset{ˆ}{\lambda }(t)$	0.2580	0.2708	0.2824	0.2864	0.2925
		(0.0028)	(0.0028)	(0.0028)	(0.0029)	(0.0026)
	${\overset{ˆ}{\lambda }}_{C}(t)$	0.2994	0.6088	0.5565	0.5176	0.4905
		(0.0093)	(0.0243)	(0.0117)	(0.0087)	(0.0072)

100	$\overset{ˆ}{\lambda }(t,p)$	1.0376	0.7503	0.6156	0.5695	0.5379
		(0.0156)	(0.0078)	(0.0045)	(0.0044)	(0.0039)
	$\overset{ˆ}{\lambda }(t)$	0.2619	0.2814	0.3040	0.3053	0.3136
		(0.0019)	(0.0033)	(0.0021)	(0.00219)	(0.0022)
	${\overset{ˆ}{\lambda }}_{C}(t)$	0.3016	0.6517	0.6176	0.5717	0.5382
		(0.0038)	(0.0127)	(0.0066)	(0.0057)	(0.0048)

**Table 5:** Mean and Mean Square Error of Hazard rate function estimators of **Gamma** distribution with exception %15 contamination **Normal error**.

		*t*				
n	Estimator	0.25	0.5	0.75	1.00	1.25

10	$\overset{ˆ}{\lambda }(t,p)$	0.1326	0.2681	0.5218	0.6577	0.8513
		(0.1697)	(0.1205)	(0.2637)	(0.3183)	(0.3111)
	$\overset{ˆ}{\lambda }(t)$	0.1352	0.2923	0.5177	0.6617	0.7996
		(0.0850)	(0.1021)	(0.1687)	(0.2667)	(0.2845)
	${\overset{ˆ}{\lambda }}_{C}(t)$	0.1237	0.2656	0.5178	0.6582	0.8010
		(0.1709)	(0.1709)	(0.2563)	(0.3234)	(0.3165)

50	$\overset{ˆ}{\lambda }(t,p)$	0.1197	0.2987	0.4772	0.6112	0.7381
		(0.0301)	(0.0358)	(0.0414)	(0.0500)	(0.0617)
	$\overset{ˆ}{\lambda }(t)$	0.1242	0.2987	0.4766	0.6128	0.7376
		(0.0151)	(0.0210)	(0.0284)	(0.0378)	(0.0533)
	${\overset{ˆ}{\lambda }}_{C}(t)$	0.1197	0.2983	0.4761	0.6889	0.7352
		(0.0305)	(0.0388)	(0.0452)	(0.0502)	(0.0651)

100	$\overset{ˆ}{\lambda }(t,p)$	0.1119	0.2856	0.4645	0.6316	0.7378
		(0.0138)	(0.0157)	(0.0118)	(0.0214)	(0.0216)
	$\overset{ˆ}{\lambda }(t)$	0.1168	0.2947	0.4662	0.6282	0.7364
		(0.0069)	(0.0093)	(0.0143)	(0.0180)	(0.0223)
	${\overset{ˆ}{\lambda }}_{C}(t)$	0.1119	0.2851	0.4630	0.6296	0.7360
		(0.0138)	(0.0176)	(0.0217)	(0.0243)	(0.0275)

**Table 6:** Mean and Mean Square Error of Hazard rate function estimators of **Gamma** distribution with exception %30 contamination **Normal error**.

		*t*				
n	Estimator	0.25	0.5	0.75	1.00	1.25

10	$\overset{ˆ}{\lambda }(t,p)$	0.1082	0.3112	0.5191	0.6436	0.7563
		(0.0687)	(0.0944)	(0.1444)	(0.1394)	(0.1496)
	$\overset{ˆ}{\lambda }(t)$	0.1240	0.3143	0.5062	0.6372	0.7511
		(0.0229)	(0.0432)	(0.0828)	(0.1036)	(0.1247)
	${\overset{ˆ}{\lambda }}_{C}(t)$	0.1089	0.3078	0.5144	0.6408	0.7564
		(0.0692)	(0.0931)	(0.1418)	(0.1365)	(0.1459)

50	$\overset{ˆ}{\lambda }(t,p)$	0.1213	0.3054	0.4833	0.6169	0.7348
		(0.0185)	(0.0254)	(0.0258)	(0.0266)	(0.0322)
	$\overset{ˆ}{\lambda }(t)$	0.1263	0.3044	0.4772	0.6143	0.7306
		(0.0058)	(0.0111)	(0.0157)	(0.0200)	(0.0274)
	${\overset{ˆ}{\lambda }}_{C}(t)$	0.1213	0.3034	0.4803	0.6115	0.7289
		(0.0185)	(0.0253)	(0.0256)	(0.0265)	(0.0335)

100	$\overset{ˆ}{\lambda }(t,p)$	0.1092	0.2975	0.4737	0.6153	0.7354
		(0.0087)	(0.0116)	(0.0137)	(0.0166)	(0.0177)
	$\overset{ˆ}{\lambda }(t)$	0.1168	0.2947	0.4709	0.6135	0.7321
		(0.0026)	(0.0056)	(0.0081)	(0.0124)	(0.0143)
	${\overset{ˆ}{\lambda }}_{C}(t)$	0.1091	0.2966	0.4706	0.6105	0.7297
		(0.0086)	(0.0115)	(0.0136)	(0.0163)	(0.0179)

**Table 7:** Mean and Mean Square Error of Hazard rate function estimators of **Gamma** distribution with exception %15 contamination **Logistics error**.

		*t*				
n	Estimator	0.25	0.5	0.75	1.00	1.25

10	$\overset{ˆ}{\lambda }(t,p)$	0.6562	0.6746	0.6560	0.7145	0.7652
		(1.9074)	(1.9076)	(0.9189)	(0.8513)	(0.7712)
	$\overset{ˆ}{\lambda }(t)$	0.3208	0.5380	0.5451	0.5512	0.5633
		(1.6052)	(9.7921)	(4.2944)	(1.2561)	(1.0152)
	${\overset{ˆ}{\lambda }}_{C}(t)$	0.1920	0.6145	0.6375	0.4997	0.6512
		(2.2239)	(1.3283)	(0.6280)	(1.1011)	(1.2015)

50	$\overset{ˆ}{\lambda }(t,p)$	0.5865	0.5795	0.5312	0.5811	0.5211
		(0.0963)	(0.0403)	(0.0285)	(0.0312)	(0.0615)
	$\overset{ˆ}{\lambda }(t)$	0.4152	0.4511	0.5121	0.5512	0.5765
		(0.1251)	(3.0145)	(2.1561)	(0.7521)	(0.8152)
	${\overset{ˆ}{\lambda }}_{C}(t)$	0.1569	0.4846	0.5466	0.5612	0.5410
		(0.0089)	(0.0138)	(0.0284)	(0.0325)	(0.0375)

100	$\overset{ˆ}{\lambda }(t,p)$	0.5663	0.5612	0.5179	0.4676	0.4713
		(0.0458)	(0.0188)	(0.0127)	(0.0115)	(0.0075)
	$\overset{ˆ}{\lambda }(t)$	0.1258	0.4715	0.5217	0.5676	0.5701
		(0.0152)	(0.5251)	(1.2561)	(1.0125)	(1.0035)
	${\overset{ˆ}{\lambda }}_{C}(t)$	0.1499	0.4672	0.5345	0.5613	0.5741
		(0.0039)	(0.0145)	(0.0129)	(0.0135)	(0.0271)

**Table 8:** Mean and Mean Square Error of Hazard rate function estimators of **Gamma** distribution with exception %30 contamination **Logistics error**.

		*t*				
n	Estimator	0.25	0.5	0.75	1.00	1.25

10	$\overset{ˆ}{\lambda }(t,p)$	0.7009	0.4799	0.3661	0.3453	0.3218
		(0.0296)	(0.0156)	(0.0090)	(0.0081)	(0.0057)
	$\overset{ˆ}{\lambda }(t)$	0.1675	0.1786	0.1779	0.1877	0.1895
		(0.0041)	(0.0044)	(0.0042)	(0.0044)	(0.0036)
	${\overset{ˆ}{\lambda }}_{C}(t)$	0.1734	0.3725	0.3488	0.3348	0.3213
		(0.0169)	(0.0363)	(0.0179)	(0.0204)	(0.0175)

50	$\overset{ˆ}{\lambda }(t,p)$	0.6995	0.5056	0.4105	0.3903	0.3639
		(0.0142)	(0.0056)	(0.0032)	(0.0027)	(0.0025)
	$\overset{ˆ}{\lambda }(t)$	0.1556	0.1675	0.1798	0.1892	0.1911
		(0.0012)	(0.0013)	(0.0012)	(0.0012)	(0.0013)
	${\overset{ˆ}{\lambda }}_{C}(t)$	0.1669	0.3800	0.3878	0.3815	0.3572
		(0.0014)	(0.0058)	(0.0036)	(0.0033)	(0.0030)

100	$\overset{ˆ}{\lambda }(t,p)$	0.6844	0.5283	0.4374	0.4190	0.3943
		(0.0100)	(0.0039)	(0.0027)	(0.0024)	(0.0027)
	$\overset{ˆ}{\lambda }(t)$	0.1437	0.1603	0.1760	0.1866	0.1918
		(0.0007)	(0.0007)	(0.0009)	(0.0009)	(0.0009)
	${\overset{ˆ}{\lambda }}_{C}(t)$	0.1671	0.4206	0.4253	0.4161	0.3950
		(0.0009)	(0.0033)	(0.0030)	(0.0027)	(0.0022)

**Table 9:** Mean and Mean Square Error of Hazard rate function estimators of **Lindley** distribution with exception %15 contamination **Normal error**.

		*t*				
n	Estimator	0.25	0.5	0.75	1.00	1.25
10	$\overset{ˆ}{\lambda }(t,p)$	2.7783	3.2788	5.3471	6.7835	8.7729
		(7.8285)	(3.7071)	(5.5905)	(3.6813)	(4.6441)
	$\overset{ˆ}{\lambda }(t)$	2.9782	4.2607	5.1686	5.4262	5.7512
		(11.2853)	(12.1825)	(13.7444)	(14.1821)	(15.0125)
	${\overset{ˆ}{\lambda }}_{C}(t)$	2.7083	2.8210	2.8808	2.9945	3.0728
		(7.0043)	(8.0775)	(10.2198)	(9.4938)	(3.1137)

50	$\overset{ˆ}{\lambda }(t,p)$	2.4261	2.5822	2.5643	2.9193	4.2800
		(1.1100)	(1.2292)	(1.4280)	(4.0251)	(7.0151)
	$\overset{ˆ}{\lambda }(t)$	2.4035	2.7237	2.5850	2.9314	3.5931
		(9.5630)	(9.6505)	(1.0931)	(10.6109)	(25.1754)
	${\overset{ˆ}{\lambda }}_{C}(t)$	2.4351	2.5812	2.9500	3.1865	3.8599
		(1.1106)	(1.1987)	(1.4599)	(3.5816)	(5.3615)

100	$\overset{ˆ}{\lambda }(t,p)$	2.3806	2.4705	2.4715	2.6567	2.9232
		(0.5221)	(0.5607)	(0.6652)	(1.1747)	(3.7315)
	$\overset{ˆ}{\lambda }(t)$	2.3813	2.4759	2.5845	2.6722	3.1407
		(0.3834)	(0.2709)	(0.4678)	(0.9517)	(1.0325)
	${\overset{ˆ}{\lambda }}_{C}(t)$	2.3779	2.4776	2.6123	2.7429	4.3145
		(0.5228)	(0.5724)	(0.7128)	(1.4172)	(1.5102)

**Table 10:** Mean and Mean Square Error of Hazard rate function estimators of **Lindley** distribution with exception %30 contamination **Normal error**.

		*t*				
n	Estimator	0.25	0.5	0.75	1.00	1.25

10	$\overset{ˆ}{\lambda }(t,p)$	2.5526	2.9583	5.3604	7.5059	7.8571
		(3.3042)	(8.3465)	(20.1521)	(271526)	(30.1526)
	$\overset{ˆ}{\lambda }(t)$	2.6554	3.2773	4.9396	3.6457	6.2025
		(2.1424)	(2.9019)	(38.3145)	(43.1197)	(55.1251)
	${\overset{ˆ}{\lambda }}_{C}(t)$	2.5398	2.8855	3.8926	3.6145	4.0920
		(3.2184)	(4.2150)	(11.2571)	(23.6092)	(27.1256)

50	$\overset{ˆ}{\lambda }(t,p)$	2.4597	2.2453	2.5706	2.6893	2.9994
		(0.5757)	(0.4811)	(0.7209)	(1.3146)	(5.1206)
	$\overset{ˆ}{\lambda }(t)$	2.4291	2.4569	2.5675	2.8085	3.1501
		(0.2721)	(0.2309)	(0.5006)	(6.0962)	(8.8652)
	${\overset{ˆ}{\lambda }}_{C}(t)$	2.4523	2.4759	2.6846	3.9638	4.9036
		(0.5785)	(0.5388)	(1.0260)	(4.1859)	(7.2352)

100	$\overset{ˆ}{\lambda }(t,p)$	2.2394	2.4187	2.5700	2.5327	2.5457
		(0.2629)	(0.2444)	(0.3007)	(0.4846)	(0.6695)
	$\overset{ˆ}{\lambda }(t)$	2.3839	2.4238	2.4938	2.5471	2.5763
		(0.1173)	(0.1200)	(0.2069)	(0.4465)	(0.6565)
	${\overset{ˆ}{\lambda }}_{C}(t)$	2.3805	2.4306	2.5885	2.7934	3.7031
		(0.2663)	(0.2743)	(1.4160)	(1.4025)	(3.5901)

**Table 11:** Mean and Mean Square Error of Hazard rate function estimators of **Lindley** distribution with exception %15 contamination **Logistics error**.

		*t*				
n	Estimator	0.25	0.5	0.75	1.00	1.25

10	$\overset{ˆ}{\lambda }(t,p)$	5.5147	4.1164	5.3217	1.8223	3.7503
		(7.5851)	(9.0335)	(12.0327)	(16.8605)	(17.7405)
	$\overset{ˆ}{\lambda }(t)$	6.6882	2.9759	3.2995	4.9049	5.2917
		(21.0421)	(7.9037)	(18.7152)	(17.3166)	(17.8526)
	${\overset{ˆ}{\lambda }}_{C}(t)$	6.3830	3.2546	1.3838	2.2360	1.2752
		(25.1752)	(9.0161)	(13.3125)	(18.1163)	(19.9394)

50	$\overset{ˆ}{\lambda }(t,p)$	2.5527	2.6978	2.3386	2.8676	3.2916
		(24.6818)	(30.6719)	(53.8453)	(52.0771)	(62.9917)
	$\overset{ˆ}{\lambda }(t)$	6.8119	1.8983	3.7467	1.8352	3.1257
		(25.5282)	(19.4680)	(21.0940)	(8.7851)	(18.5015)
	${\overset{ˆ}{\lambda }}_{C}(t)$	4.6538	6.0101	7.1852	9.6125	10.1055
		(7.8405)	(8.8452)	(8.9165)	(9.1252)	(8.9521)

100	$\overset{ˆ}{\lambda }(t,p)$	2.1867	2.0712	2.1926	3.2887	2.6668
		(4.2219)	(4.9383)	(7.6555)	(9.6982)	(10.1251)
	$\overset{ˆ}{\lambda }(t)$	2.1514	2.4450	2.2235	5.0192	2.3625
		(8.0762)	(11.8992)	(7.1313)	(10.1521)	(7.9125)
	${\overset{ˆ}{\lambda }}_{C}(t)$	3.1412	2.7614	1.2032	1.0316	0.8924
		(1.4215)	(1.3039)	(1.1253)	(8.0082)	(6.9522)

**Table 12:** Mean and Mean Square Error of Hazard rate function estimators of **Lindley** distribution with exception %30 contamination **Logistics error**.

		*t*				
n	Estimator	0.25	0.5	0.75	1.00	1.25

10	$\overset{ˆ}{\lambda }(t,p)$	1.3312	0.8408	0.6514	0.5856	0.5686
		(0.1313)	(0.0614)	(0.0394)	(0.0445)	(0.0424)
	$\overset{ˆ}{\lambda }(t)$	0.4405	0.4519	0.4553	0.4338	0.4442
		(0.1287)	(0.1728)	(0.2093)	(0.0766)	(0.0721)
	${\overset{ˆ}{\lambda }}_{C}(t)$	2.2371	3.4598	2.2089	0.9798	3.4464
		(1.4202)	(1.8752)	(1.0301)	(1.2515)	(1.0418)

50	$\overset{ˆ}{\lambda }(t,p)$	1.2713	0.8012	0.6045	0.5225	0.5142
		(0.0224)	(0.0088)	(0.0057)	(0.0042)	(0.0041)
	$\overset{ˆ}{\lambda }(t)$	0.3675	0.3787	0.3819	0.3809	0.3828
		(0.0038)	(0.0037)	(0.0036)	(0.0035)	(0.0033)
	${\overset{ˆ}{\lambda }}_{C}(t)$	2.9368	1.0193	0.7997	0.6719	0.6201
		(0.0914)	(1.3206)	(0.9151)	(0.3517)	(0.2512)

100	$\overset{ˆ}{\lambda }(t,p)$	1.2799	0.7957	0.5991	0.5501	0.5074
		(0.0118)	(0.0042)	(0.0027)	(0.0024)	(0.0022)
	$\overset{ˆ}{\lambda }(t)$	0.3689	0.3734	0.3761	0.3780	0.3774
		(0.0019)	(0.0019)	(0.0016)	(0.0016)	(0.0017)
	${\overset{ˆ}{\lambda }}_{C}(t)$	2.4997	1.5626	0.7571	0.6547	0.5941
		(0.0125)	(1.2562)	(0.0237)	(0.0133)	(0.0116)

Black line: Actual value, Blue line: Estimator (1.12), Red line: Estimator (1.7), and Green line: Estimator (4.1).

As observed from these results, our proposed estimators have generally outperformed the estimator (4.1) in terms of both empirical mean square error and bias, especially for large *t*. The results revealed that with a large sample size, the improvement of the proposed estimators would be further enhanced. Comparison between our two proposed estimators indicates the local polynomial estimator in terms of empirical mean square error is better than the first plug-in estimator.

## 5 Conclusion

Considering the importance of the hazard rate function in the evaluation of survival data, its point estimation with the presence of measurement errors generated from normal distribution is considered. In using the deconvolution kernel density function estimator, we proposed a plug-in estimator. The second estimator was constructed using the first estimator and applying local polynomial regression. For measurement errors that have a normal or logistic distribution, we mentioned the conditions under which our proposed plug-in estimator is consistent. For the local polynomial estimator, the asymptotic bias and variance were calculated and noted that this estimator has also an asymptotic normal distribution. Note that the mean square error of the statistical distribution of the survey with the logistic distribution error was lower compared to the mean square of the error with the normal distribution error under the same conditions.

## CRediT authorship contribution statement

**Parviz Nasiri:** Supervision, Methodology. **Rougheih Kheirazar:** Software, Investigation. **Abbas Rasouli:** Software. **Ali Shadrokh:** Resources.

## Declaration of Competing Interest

The authors declare the following financial interests/personal relationships which may be considered as potential competing interests:

Parviz Nasiri reports a relationship with Payame Noor University that includes: non-financial support. Parviz Nasiri has patent licensed to –. It is declared that there is no conflict of interest. Parviz Nasiri

If there are other authors, they declare that they have no known competing financial interests or personal relationships that could have appeared to influence the work reported in this paper.

## Data Availability

Data will be made available on request.
